# Research Progress on the Treatment of Geriatric Intertrochanteric Femur Fractures with Proximal Femur Bionic Nails (PFBNs)

**DOI:** 10.1111/os.14134

**Published:** 2024-07-09

**Authors:** Wenyu Duan, Hao Liang, Xiaolong Fan, Dongming Zhou, Yulu Wang, Haidong Zhang

**Affiliations:** ^1^ Baotou Medical College Baotou China; ^2^ Department of Traumatic Surgery, The First Affiliated Hospital of Baotou Medical College Baotou China

**Keywords:** Intertrochanteric femur fractures, Proximal femoral nail anti‐rotation, Proximal femur bionic nail

## Abstract

Intertrochanteric femur fracture is the most common hip fracture in elderly people, and the academic community has reached a consensus that early surgery is imperative. Proximal femoral nail anti‐rotation (PFNA) and InterTan are the preferred internal fixation devices for intertrochanteric femur fractures in elderly individuals due to their advantages, such as a short lever arm, minimal stress shielding, and resistance to rotation. However, PFNA is associated with complications such as nail back‐out and helical blade cut‐out due to stress concentration. As a new internal fixation device for intertrochanteric femur fractures, the proximal femoral biodegradable nail (PFBN) addresses the issue of nail back‐out and offers more stable fracture fixation, a shorter lever arm, and stress distribution compared to PFNA and InterTan. Clinical studies have shown that compared to PFNA, PFBNs lead to faster recovery of hip joint function, shorter non‐weight‐bearing time, and faster fracture healing. This article provides a literature review of the structural characteristics, biomechanical analysis, and clinical studies of PFBNs, aiming to provide a theoretical basis for the selection of internal fixation devices for the treatment of intertrochanteric femur fractures in elderly patients and to improve the quality of life of patients during the postoperative period.

## Graphical abstract

This article presents a comprehensive narrative review of literature pertinent to proximal femur bionic nails (PFBN), meticulously sourced from PubMed and other prominent databases. The review is structured around three pivotal facets: the structural characteristics of PFBN, its biomechanical dynamics, and clinical research findings. Notably, PFBN exhibits superior mechanical advantages compared to both InterTan and PFNA, an assertion that has been substantiated by clinical experimental data.

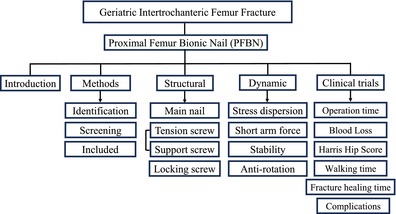

## Introduction

With the development of society and the economy, the incidence of low‐energy hip fractures caused by osteoporosis in elderly individuals is increasing annually.^1^, ^2^ Among hip fracture patients aged 75 and above, the mortality rate for intertrochanteric fractures is greater than that for femoral neck fractures.^3^, ^4^ The academic community has reached a consensus on the importance of early surgical treatment for geriatric intertrochanteric fractures. It can effectively reduce the incidence of complications such as pressure ulcers, deep vein thrombosis, pulmonary embolism, aspiration pneumonia, and urinary tract infections. Additionally, it significantly reduces the risk of death within 24 months after injury.^5^, ^6^

Proximal femoral nail anti‐rotation (PFNA) and InterTan are the leading treatment options for elderly patients with intertrochanteric fractures due to their short lever arm, minimal stress shielding, and anti‐rotational capabilities. However, complications such as stress concentration, blade cut‐out, and nail back‐out can occur, necessitating careful surgical planning to ensure the best patient outcomes.^7^, ^8^, ^9^ Compared to PFNA, InterTan offers improved anti‐rotational stability and a lower incidence of resection and screw migration complications. However, it is important to note that there is still a risk of postoperative complications such as internal fixation failure and the development of coxa vara. PFBNs has designed to solve problems. Compared to PFNA, PFBNs offer better maintenance of fracture stability, reduced stress concentration, prevention of cut‐out, and avoidance of nail back‐out.^10^, ^11^

**FIGURE 1:**
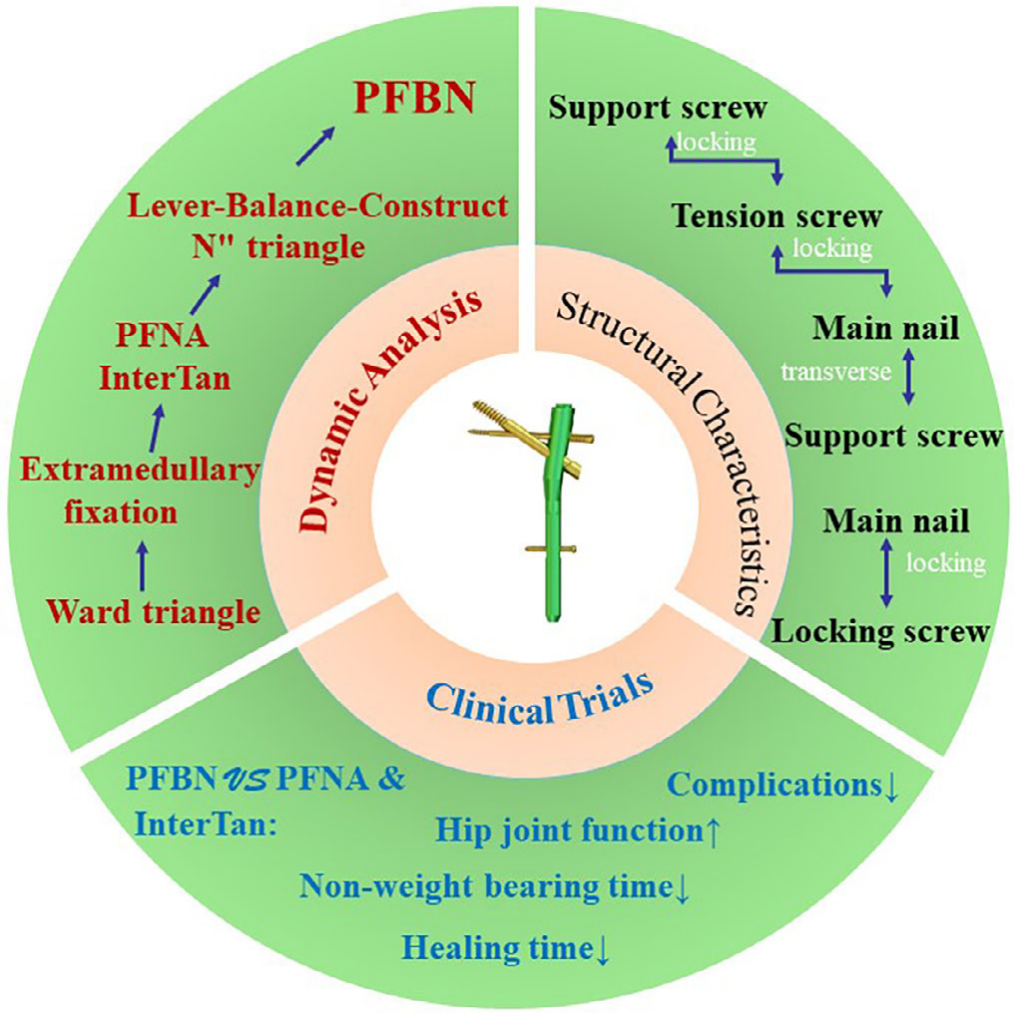
Proximal femur bionic nail (PFBN) include structural characteristics, dynamic analysis, and clinical trials.

Therefore, This article provides a literature review of the design features, mechanical characteristics, and clinical studies of PFBNs, aiming to provide a theoretical basis for surgical decision‐making in the treatment of geriatric intertrochanteric fractures (Figure 1).

## Methods

**FIGURE 2:**
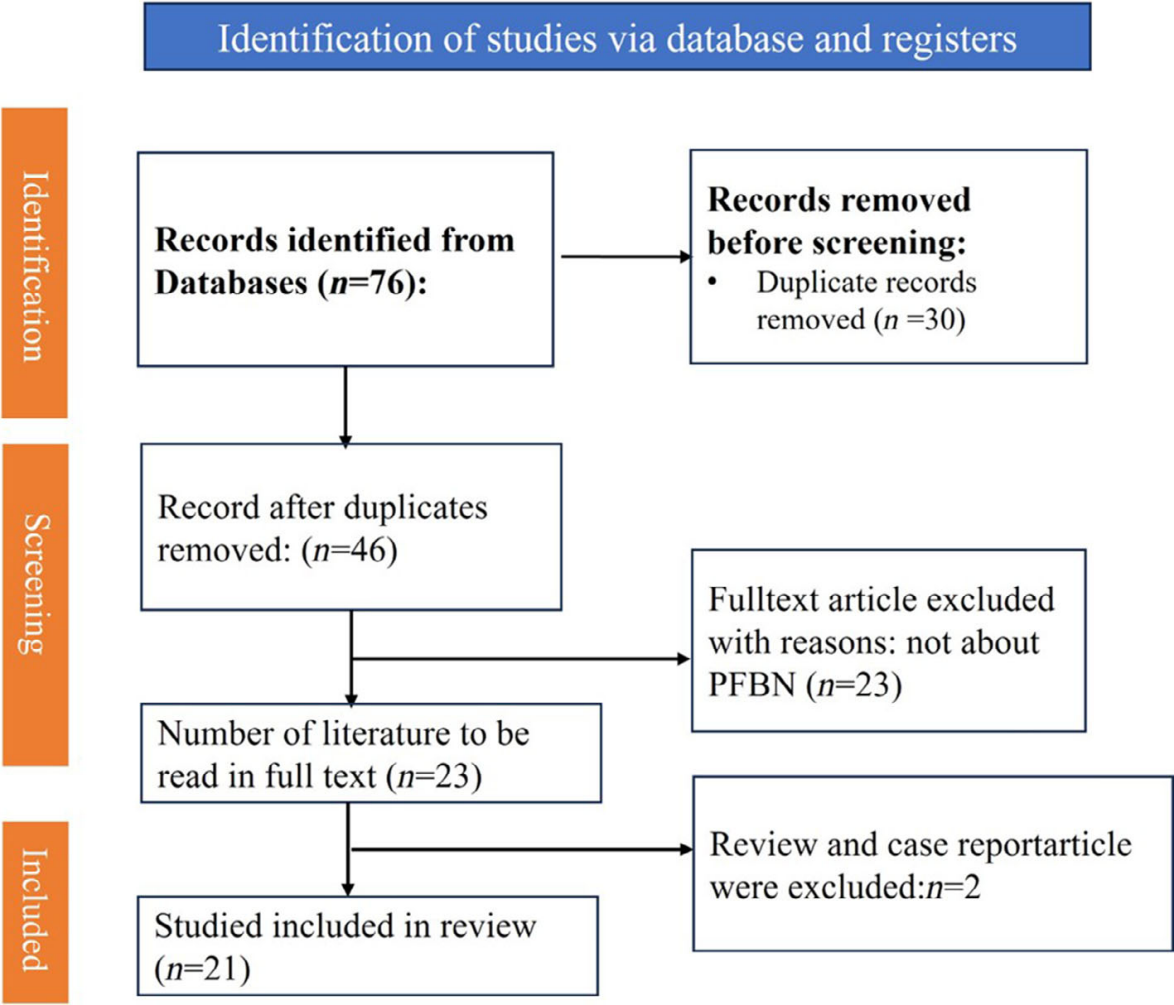
Preferred reporting items for proximal femur bionic nail (PFBN) articles flowchart displaying the search and selection process performed.

A comprehensive literature search for articles published between the inception to January 1, 1997 was performed using PubMed, Google scholar, Embase, and Web of Science. The following combination of keywords and MeSH terms were used: (Proximal Femur Bionic Nail) OR (PFBN). A total of 76 publications were retrieved. Two review and case report articles, 30 duplicated articles, and 23 articles unrelated to “Proximal Femur Bionic Nail” were excluded. Eventually, 21 articles were included to review. The quality assessment of included literature was independently conducted by two authors. The Jadad scale (modified version) was used for evaluating randomized controlled trials, while the Newcastle–Ottawa scale (NOS) was applied for retrospective case studies.^6^, ^12^, ^13^ In the event of discrepancies, a third author was consulted, and consensus was reached through discussion. There are a total of 21 articles related to PFBN, out of which 12 focus on the biomechanical analysis of PFBN and are almost all indexed by SCI. Nine articles are clinical case–control studies, and we conducted a literature quality evaluation on these nine articles. (Table 1, Figure 2).

**TABLE 1:** Summary of the clinical research literature on PFBN, PFNA, and InterTan.

Study year	Group	Sample size	Mean age, years	Operation time (minutes)	Intraoperative blood loss (mL)	Harris score	Weight‐ bearing time (day)	Fracture healing time (month)	NOS/Jadad score
Ling F, 2023	PFBN	28	70.4 ± 7.8	57.9 ± 12.0	138.28 ± 6.27	83.9 ± 4.3	‐	15.6 ± 3.6	8
	PFNA	28	73.0 ± 8.9	59.6 ± 10.6	115.79 ± 5.69	81.0 ± 3.4	‐	18.8 ± 4.8	
Li ZT, 2022	PFBN	46	75.7 ± 5.2	47.3 ± 11.4	130.6 ± 21.3	‐	7.9 ± 2.7	12.3 ± 0.5	7
	PFNA	46	75.3 ± 4.2	39.2 ± 15.3	123.5 ± 17.8	‐	21.2 ± 5.7	12.6 ± 0.7	
Yang DS, 2023^39^	PFBN	24	79.0 ± 5.0	39.25 ± 8.28	110.00 ± 17.69	‐	7.9 ± 1.7	12.38 ± 2.55	8
	PFNA	24	78.6 ± 5.8	27.00 ± 6.21	105.00 ± 14.74	‐	17.63 ± 3.7	12.29 ± 2.03	
Liu PY, 2021^42^	PFBN	35	71.81 ± 7.63	45.66 ± 8.73	93.73 ± 16.26	89.18 ± 10.2	10.74 ± 2.19	17.13 ± 2.34	6
	PFNA	37	72.33 ± 6.47	43.31 ± 7.68	98.66 ± 20.63	95.63 ± 10.8	12.21 ± 2.26	19.56 ± 3.78	
Wang YC, 2023	PFBN	20	75.3 ± 6.4	55.9 ± 8.2	35.2 ± 9.9	80.4 ± 4.4	15.8 ± 1.4	‐	7
	PFNA	20	74.6 ± 6	45.4 ± 7.9	34.8 ± 7.9	79.4 ± 4.3	55.3 ± 3.3	‐	
Fu HP, 2023	PFBN	18	76.0 ± 4.8	70.00 ± 7.9	‐	‐	5.7 ± 1.2	14.5	7
	PFNA	36	79.7 ± 7.8	60.30 ± 6.6	‐	‐	21.5 ± 3.6	15.5	
	InterTan	14	81.4 ± 7.6	61.0 ± 5.0	‐		14.4 ± 2.1	16	
Jin LK, 2024	PFBN	25	73.67 ± 5.16	81.14 ± 12.65	179.81 ± 40.09	70.52 ± 5.34	7.98 ± 1.34	10.14 ± 2.33	6
	PFNA	55	74.23 ± 5.57	74.10 ± 8.62	172.84 ± 21.08	51.46 ± 5.36	10.27 ± 0.66	13.68 ± 2.36	
	InterTan	40	73.45 ± 5.34	81.62 ± 8.56	181.65 ± 23.58	69.81 ± 6.17	8.22 ± 0.46	11.87 ± 2.48	
Wan Y, 2024	PFBN	16	78.0 ± 8.8	111.6 ± 15.9	145.6 ± 39.5	64.9 ± 5.4	19.1 ± 2.0	‐	7
	InterTan	19	75.3 ± 7.0	98.9 ± 15.9	120.5 ± 31.1	55.0 ± 5.7	23.8 ± 3.2	‐	
Sun ZH, 2024	PFBN	26	74.92 ± 9.31	64.65 ± 21.37	‐	74.81 ± 11.20	33.39 ± 8.89	‐	8
	Hip	30	80.63 ± 6.73	68.90 ± 23.80	‐	62.64 ± 25.48	9.87 ± 2.12	‐	

Abbreviations: NOS, Newcastle‐Ottawa scale; PFBN, proximal femoral biodegradable nail; PFNA, proximal femoral nail anti‐rotation.

## Structural Characteristics

**FIGURE 3:**
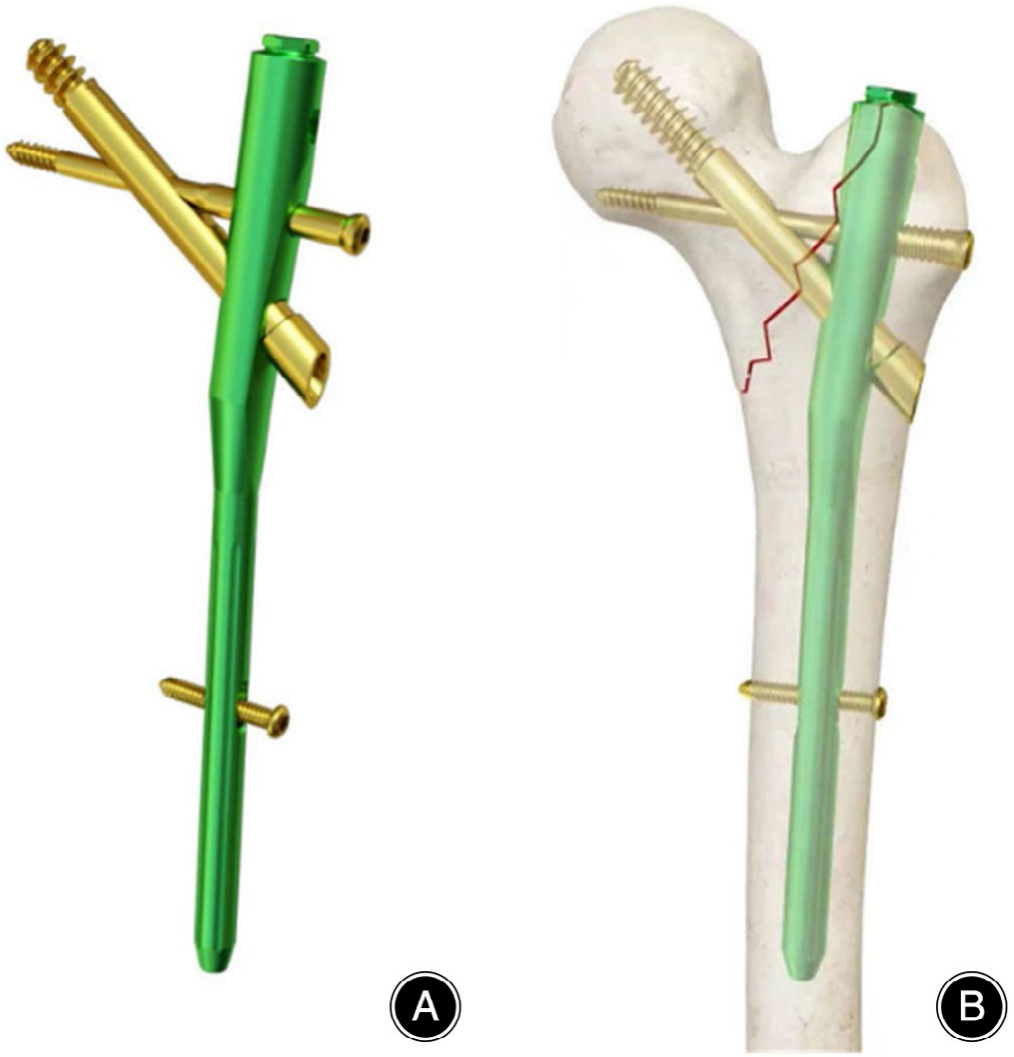
(A) Proximal femur bionic nail (PFBN) (B) Illustration of a PFBN inserted into the fracture ends.^16^

**FIGURE 4:**
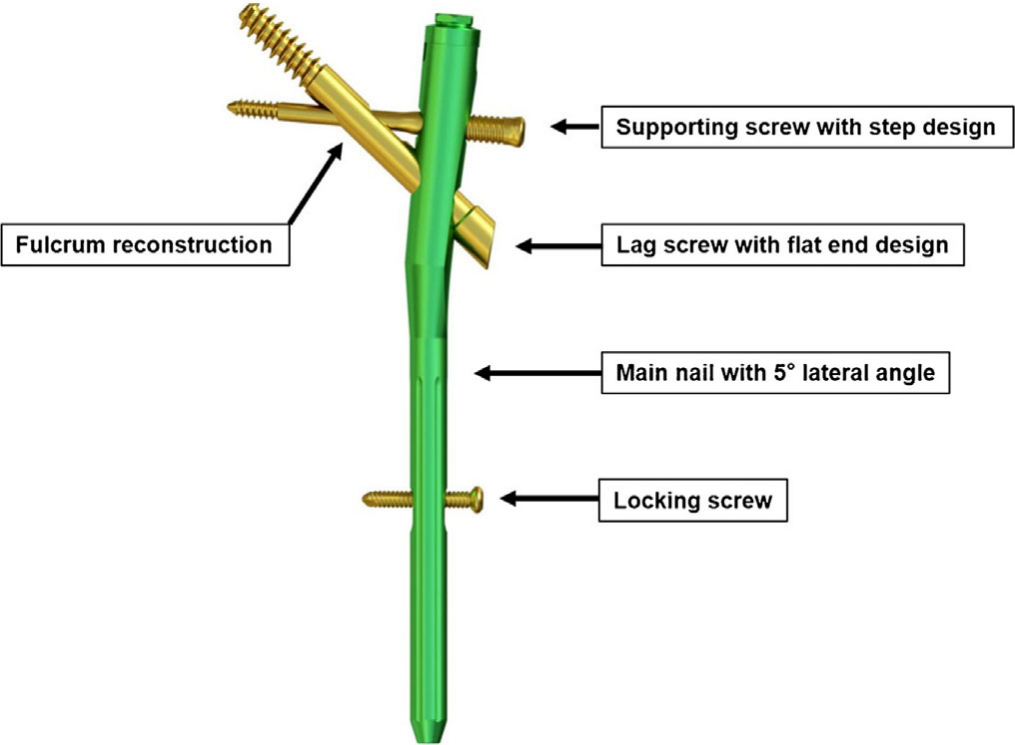
Schematic diagram of the components of a proximal femur bionic nail (PFBN).^16^

PFBNs are composed of four components: a main nail, a tension screw, a transverse support screw, and a locking screw. The main nail has three nail holes designed to accommodate the tension screw, support screw, and distal locking screw. The tension screw has an additional nail hole for the insertion of the support screw. The proximal end of the main nail is angled outward by 5°, and the nail hole for the support screw on the main nail has internal threads, while the shaft of the support screw has external threads that match the internal threads.^14^ The front end of the tension screw features an over‐tapping hole that is inserted into the femoral head in the direction of the compressive trabeculae. The support screw passes through the nail hole of the tension screw and is inserted into the femoral head in the direction of the tensile trabeculae. Finally, the locking screw is used to fix the main nail and prevent intramedullary rotation (Figures 3 and 4).^15^ The surgical procedure involving PFBNs was similar to that for other intramedullary fixation devices, such as PFNA and InterTan, which makes it easier for the technique to be widely adopted.^13^

The proximal end of the main nail is angled outward so that it can be placed at the apex of the greater trochanter. The threaded structure of the support screw ensures a close connection between the main nail and tension screw, effectively preventing the risk of loosening, back‐out, and cut‐out of the tension screw and support screw in the femoral head. Compared to PFNA and InterTan, the support screw can effectively restore the integrity of the trabecular structures, maintaining the structural integrity of the femoral medullary canal.

## Dynamic Analysis

### The Theoretical Basis of PFBN

The proximal femur is one of the strongest bone tissues in the body. During walking, the proximal femur bears 2–3 times the body weight load (body weight, BW).^17^, ^18^ In 1838, Ward first proposed the concept of the Ward triangle, which is formed by the intersection of the medial trabecular system and the lateral trabecular system in the proximal femur. Henke and Koch further improved this theory and noted that the cantilever structure is the standard mechanical structure for the treatment of intertrochanteric femur fractures.^19^, ^20^ However, complications such as screw fracture, removal, and hip internal rotation due to off‐center fixation, long lever arms, and highly concentrated stresses led to the development of intramedullary fixation devices such as PFN, PFNA, and InterTan.^21^, ^22^

**FIGURE 5:**
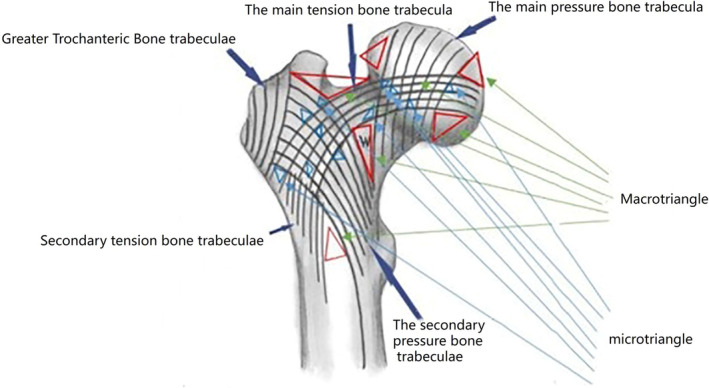
Schematic diagram of the coronal structure of the upper femur: a number of macro‐triangles can be observed, such as red triangle tension trabecula, main pressure trabecula, Ward triangles and the inner and lower boundary of the femoral head. Since the two types of bone trabeculae cross each other, at the microscopic level, the proximal femur is composed of countless tension trabeculae and pressure trabeculae forming micro‐triangles (blue triangles).^11^

Recently, Xu *et al*. found that the triangular structure formed by the medial wall, lateral wall, and upper wall of the proximal femur bears the main stress. Poor reduction of the integrity of the medial wall during fracture surgery is an important cause of postoperative complications related to fixation failure. This concept is also consistent with the findings of Fan *et al*.^21^, ^23^ Zhang *et al*. “lever‐balance‐reconstruction” theory describes the proximal femur as a lever system whose disruption by fractures impairs weight‐bearing and causes deformities. They argue that restoring the femoral fulcrum with internal fixation devices is crucial for stable postoperative outcomes, with optimal stability achieved when the fulcrum is near its natural position.^24^, ^25^ According to a retrospective analysis by Zhang *et al*., the failure of internal fixation devices in 40 patients with proximal femoral fractures was attributed to an imbalance of forces on the pressure and tension sides caused by different reconstruction pivot positions after femoral neck fracture surgery. This imbalance leads to an inability to effectively maintain the reestablished equilibrium, ultimately resulting in the failure of internal fixation devices in femoral neck fractures.^26^ Additionally, Zhang *et al*. proposed the “N” triangle theory for the proximal femur. They suggested that along with the Ward triangle, there are multiple large triangles and countless small triangles formed by the intersection of five types of trabecular structures in the proximal femur (Figure 5).^11^ When an intertrochanteric femur fracture occurs, the fracture line disrupts the stable triangular structure formed by the trabeculae, resulting in the loss of its stabilizing function.^27^

### The Dynamic Properties of PFBN

Based on the “lever‐balance‐construct” and “N” triangle theory, Zhang *et al*. conducted a study and designed a PFBN implant.^11^ The PFBN implant brings the fixed balance point closer to the medial side of the femoral neck, either in the lateral wall of the femur or in the medullary cavity, resulting in a shorter lever arm during weight‐bearing and improved stability.

In Wang *et al*.'s study,^28^ they compared the mechanical properties of three intramedullary fixation devices (PFBN, PFNA, and InterTan) using a finite element model constructed from the femur of a 65‐year‐old female (height: 168 cm, weight: 70 kg, vertical loading: 2100 N). They found that under the same stress conditions, the stress distribution on the femoral head was more dispersed, and the deformation was smaller with PFBNs than with PFNAs and InterTan. This indicates that PFBN implantation is more physiological and carries a lower risk of screw cutout from the femoral head. Additionally, due to the interlocking mechanism between tension screws and support screws, most of the stress is shared, thus reducing the risk of screw failure caused by stress concentration.^28^

The triangular plane formed by the main screw, tension screws, and support screws in PFBNs provides excellent anti‐rotation effects and is more in line with the physiological characteristics of the proximal femur, achieving biomechanical fixation of the proximal femur. In a recent study by Chen *et al*.,^29^ a finite element model was created to compare the performance of PFBNs, PFNA, and DHS as secondary internal fixation devices after PFNA blade extraction under a longitudinal force of 2100 N. Compared to extramedullary fixation with dynamic hip screw (DHS), the reimplantation of PFNA and PFBNs as intramedullary fixation resulted in more stable fracture ends and lower stress, demonstrating better treatment outcomes. Compared with those of PFBNs and PFNA, the fracture end fixation with PFBNs was more stable and had lower stress shielding. The risk of implant cut‐out was significantly reduced. Moreover, the postoperative displacement of the femoral head and internal fixation device was significantly smaller with PFBNs than with PFNA and DHS, indicating its superior biomechanical stability. Ding *et al*.^10^, ^30^ mechanical analysis of femoral neck fractures demonstrated that the PFBN provides greater cross‐sectional stability under axial stress compared to the DHS and canulated screws (CSs). This increased stability helps to reduce stress concentration, enhances the overall stability of the fracture fixation construct, and is more congruent with the tissue structure and biomechanics of the proximal femur. The research findings of Cheng *et al*. are consistent with them.^31^

## Clinical Trials

### Operation Time

Li *et al*. conducted a retrospective analysis of 92 elderly patients with intertrochanteric femur fractures and found that compared to PFNA and InterTan group, the PFBN group had a longer time to operation time(*p* < 0.05).^32^ Yang *et al*. and others research findings have pointed out this issue.^33^, ^34^, ^35^ The challenges associated with PFBN may stem from it being a relatively new internal fixation method. Surgeons may not be as proficient with this device, which can contribute to longer surgical times compared to PFNA. The addition of a supplementary compression screw between the small trochanters in PFBN procedures could further extend the duration of surgery, potentially affecting the overall efficiency and familiarity for operators.

### Intraoperative Blood Loss

In a retrospective analysis of 56 elderly patients with intertrochanteric femur fractures, Ling *et al*. found that the use of PFBN, compared to PFNA and InterTan, showed no significant differences in terms of intraoperative blood loss, consistent with findings from other studies.^32^, ^33^, ^36^ They found that closed reduction with PFBN as the internal fixation method did not inflict more harm on patients compared to alternative devices, potentially aiding a faster postoperative recovery.

### Harris Hip Score

Studies by Wang *et al*. and Jin have demonstrated that patients treated with PFBN and InterTan experienced significant improvements in Harris hip score within 1 month post‐surgery (*p* < 0.05) compared to those treated with PFNA. However, at the 6 months follow‐up and beyond, there was no significant (*p* > 0.05) difference observed.^35^, ^37^, ^38^ This showed that PFBN effectively restores hip joint function shortly after surgery. Improved hip joint mobility in the early postoperative period can also enhance the quality of life for patients and alleviate the burden on both their families and society.

### Weight‐Bearing Time

A retrospective study by Li *et al*. showed that in 92 elderly patients with intertrochanteric femur fractures, the PFBN group had a shorter non‐weight‐bearing period, enabling early weight‐bearing compared to the PFNA group (*p* < 0.05). This finding suggests that clinicians may have greater confidence in the early stability of PFBNs as an internal fixation device.^32^ Yang *et al*. conducted a retrospective study involving 48 surgical patients and found that the PFBN group not only had a shorter non‐weight‐bearing time but also achieved better outcomes in terms of postoperative VAS scores and walking ability assessment.^33^, ^39^ The use of PFBN as an internal fixation device for intertrochanteric femur fractures allows for early weight bearing, which can effectively reduce the occurrence of fatal postoperative complications in elderly patients, such as aspiration pneumonia, urinary tract infections, and pressure ulcers. However, it should be noted that adequate fracture reduction is crucial for successful surgery with PFBNs, as PFBNs provide static fixation. Poor reduction may lead to gaps that cannot be eliminated through secondary compression, resulting in delayed fracture healing or nonunion.^33^ This viewpoint was further supported by Lin *et al*., who emphasized the importance of central placement of the tension screw to avoid cut‐out of the support screw from the femoral head/neck.^40^ Li *et al*. suggested that in the selection of different internal fixation devices for elderly patients with intertrochanteric fractures, PFBN provides good initial postoperative stability, allowing for early weight‐bearing exercise and demonstrating more precise short‐term efficacy.^41^

### Fracture Healing Time

Ling *et al*. found in six studies that patients in the PFBN group had a shorter healing time for femoral intertrochanteric fractures compared to patients in the InterTan and PFNA groups.^32^, ^33^, ^34^, ^36^, ^42^ This indicated that the PFBN can more effectively maintain stability at the fracture site and promote fracture healing, which is consistent with the conclusions of biomechanics research by Wang *et al*. and Chen *et al*.^29^, ^37^

### Complications

**FIGURE 6:**
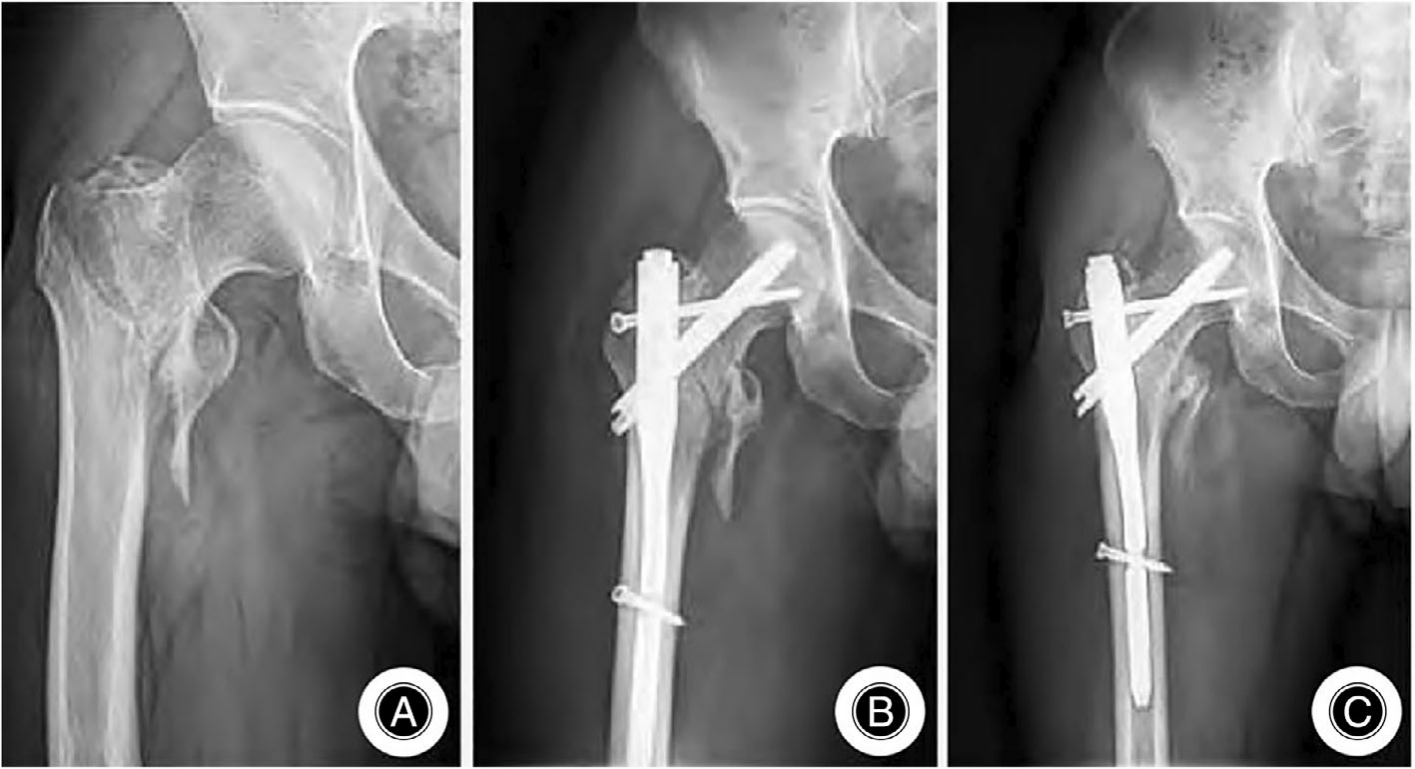
Proximal femur bionic nail (PFBN) internal fixation treatment: (A) preoperative right hip anteroposterior X‐ray showing an Evans‐Jensen IV type intertrochanteric femur fracture. (B) Immediate postoperative anteroposterior X‐ray demonstrating good fracture reduction and well‐positioned internal fixation hardware. (C) Anteroposterior X‐ray at 3 months postoperatively showing no displacement of the fracture or internal fixation hardware, with a blurred fracture line.

Ling *et al*. in four studies on postoperative complications revealed a significantly lower incidence of complications in the PFBN group compared to the PFNA group, with statistical significance observed in the study by Yang and Liu (*p* < 0.05). The PFBN group also exhibited a notably reduced rate of internal fixation failure compared to PFNA, suggesting that PFBN provides stable fracture fixation and maintains stability at the fracture ends^33^, ^36^, ^38^, ^42^ (Table 2). Hao *et al*. highlighted that hip internal rotation significantly increases proximal femoral torque in elderly patients with intertrochanteric femur fractures, leading to increased stress concentration on the internal fixation device. This increases the risk of helical blade cut‐out and internal fixation device fracture in patients treated with PFNA.^43^ The PFBN group also showed less loss of the neck‐shaft angle and a lower risk of postoperative hip internal rotation deformity than did the PFNA group (Figure 6).^37^

**TABLE 2:** Summary of the clinical research literature about complications on PFBN and PFNA, Inter Tan.

Study year	Group	Sample size	Nonunion	Loosening of the internal fixation	Lower limb venous thrombosis	Hip varus	Total
Ling F, 2023	PFBN	28		0			0
	PFNA	28		1			1
Yang DS, 2023	PFBN	24	0	0	2		2
	PFNA	24	3	3	3		9
Liu PY, 2021	PFBN	35	1	1	0		2
	PFNA	37	2	4	1		7
Jin LK, 2024	PFBN	25	0	0		1	1
	PFNA	55	1	1		2	4
	InterTan	40	0			2	2

Abbreviations: PFBN, proximal femoral biodegradable nail; PFNA, proximal femoral nail anti‐rotation.

The use of PFBN as an internal fixation device for intertrochanteric femur fractures allows for early weight bearing, which can effectively reduce the occurrence of fatal postoperative complications in elderly patients, such as aspiration pneumonia, urinary tract infections, and pressure ulcers. Improved hip joint mobility in the early postoperative period can also enhance the quality of life for patients and alleviate the burden on both their families and society. However, it should be noted that, as a relatively new fixation method, several studies have reported longer operating times for PFBN surgeries. This may be attributed to the greater requirements for fracture reduction, surgeons' unfamiliarity with the procedure, and the additional time needed for the placement of the support screw.^32^, ^34^, ^37^, ^39^

## Conclusion

In summary, PFBN demonstrates promising prospects in finite element analysis due to its mechanical stability, lower stress shielding, and rotational resistance. Clinical studies also indicate that PFBN can lead to earlier recovery of hip joint function, shorter non‐weight‐bearing time, and improved postoperative quality of life. However, PFBN requires better fracture reduction and placement of the support screw, which may prolong the surgical duration and potentially increase the risks from anesthesia and of postoperative infection. Currently, there is limited high‐quality literature available on the clinical application of PFBN. This review that the use of PFBN as an internal fixation device for intertrochanteric femur fractures is more closely related to the biomechanics of the proximal femur, offering advantages in terms of fracture healing, improving quality of life, and reducing surgical complications compared to other fixation methods. We hope that this review will encourage the academic community to pay greater attention to the use of PFBN as an emerging internal fixation device for intertrochanteric femur fractures in elderly people and stimulate more extensive and in‐depth research in this field.

## Conflict of Interest Statement

All the authors declare that they have no conflict of interest.

## Ethics Statement

None.

## Author Contributions

DY and LH contribute equally to this manuscript. WY has contributed to the conception, study design, and drafting of the study. LH, FL, and ZM have contributed to the acquisition, analysis, and interpretation of the data. DW, LH, FL, ZM, and ZD have revised the manuscript. All authors have approved the final version of the manuscript and have agreed to be personally accountable for the author's contributions and questions related to the accuracy or integrity of any part of the work.

## Supporting information


**Data S1.** Supporting Information.
