# Supplementary material for: Research Progress on the Treatment of Geriatric Intertrochanteric Femur Fractures with Proximal Femur Bionic Nails (PFBNs)
Source: Orthop Surg. 2024 Jul 9;16(10):2303–10. doi: 10.1111/os.14134 (PMC11456711; doi:10.1111/os.14134)
Supplement: Supplementary file 1 — Data S1. Supporting Information. [file OS-16-2303-s001.pdf]

内蒙古自治区教育厅

ᠥᠪᠥᠷ ᠮᠣᠩᠭᠣᠯ ᠤᠨ ᠥᠪᠡᠷᠲᠡᠭᠡᠨ ᠵᠠᠰᠠᠬᠤ ᠣᠷᠣᠨ ᠤ ᠪᠣᠯᠪᠠᠰᠤᠷᠠᠯ ᠤᠨ ᠲᠢᠩᠭ

内教研函〔2022〕8号

关于下达2021年全区研究生科研创新项目的通知

各研究生培养单位：

根据《内蒙古自治区研究生科研创新项目管理办法（试行）》（以下简称《办法》）有关规定，教育厅在各单位遴选推荐的基础上，组织专家对报送的项目进行了形式审查和总体把关。经审核，决定对“数字技术融合与企业环境绩效——基于技术环境不确定性视角”等88项博士研究生科研创新项目和“高校教师职业倦怠成因的定性比较分析——工作要求-资源模型视角”等187项硕士研究生科研创新项目予以资助，现予下达。

各单位按《办法》要求做好项目资金配套和实施管理，于2023年3月底前向自治区教育厅提出验收申请，并将结题材料（纸质版、电子版各一份）统一报送至自治区教育厅学位管理与研究生教育处。

电子邮箱：nmg_xwb@163.com

地　　址：自治区教育厅学位管理与研究生教育处

呼和浩特市新城区丁香路5号601室

邮　　编：010011

联 系 人：马辉　　　　　联系电话：0471-2856627

附件：1.内蒙古自治区 2021 年博士研究生科研创新项目汇总表

2.内蒙古自治区 2021 年硕士研究生科研创新项目汇总表

内蒙古自治区教育厅

2022年3月10日

46	内蒙古科技大学	S20210153Z	李晓东	硕士	2020级	虞启辉	基于喷雾传热技术的压缩空气准等温膨胀过程研究	自然科学	机械工程
47	内蒙古科技大学	S20210154Z	徐传帅	硕士	2020级	赵宇红	基于稀疏卷积神经网络的种子分类方法研究	自然科学	计算机科学与技术
48	内蒙古科技大学	S20210155Z	施鑫垚	硕士		王静宇	基于区块链的数据共享技术研究	自然科学	电子信息（计算机技术）计算机科学
49	内蒙古科技大学	S20210156Z	赵东波	硕士	2020级	李科	用于光电领域的高性能铈基稀土抛光粉开发	自然科学	动力工程及工程热物理
50	内蒙古科技大学	S20210157Z	何泓江	硕士	2020级	赫文秀	磷酸锰锂@碳/石墨烯纳米复合材料的合成	自然科学	化学工程与技术
51	内蒙古科技大学	S20210158Z	李晓艺	硕士	2021级	孔敬	内蒙古东部地区历史城池空间发展研究	自然科学	建筑学
52	内蒙古科技大学	S20210159Z	付哲	硕士	2020级	邓磊波	堇青石透明玻璃陶瓷的析晶行为及性能研究	自然科学	物理学
53	内蒙古科技大学	S20210160Z	蔺思函	硕士	2020级	杜志强	多巴胺受体与多巴脱羧酶协同介导克氏原螯虾抗细菌先天免疫反应的分子机制	自然科学	生物学
54	内蒙古科技大学	S20210161Z	吕楠楠	硕士	2020级	张鹏	基于神经科学的社交媒体情境下消费者冲动购买机制研究	自然科学	管理科学与工程
55	内蒙古科技大学	S20210162Z	程文静	硕士	2020级	杨哲	党百年来坚守人民立场的内在逻辑研究	人文社科	马克思主义理论
56	内蒙古科技大学	S20210163Z	陈□娟	硕士	2021级	韩冬楠	内蒙古沿□地区传统□艺谱系构建与创新设计研究	人文社科	设计学
57	内蒙古科技大学	S20210164Z	王聪慧	硕士	2021级	朱海珅	乡村振兴战略下宅基地流转问题的调研报告	人文社科	法律
58	内蒙古科技大学	S20210165Z	郭鑫伟	硕士	2020级	李建伟	基于三维数值重构的岩石破裂裂隙形成机理研究	自然科学	矿业工程
59	内蒙古科技大学包头医学院	S20210174Z	王冠	硕士	2020级	耿立霞	MCP通过Gal-3影响慢性同种异体移植物损伤导致的肾纤维化	自然科学	临床医学（学术学位）
60	内蒙古科技大学包头医学院	S20210175Z	段文禹	硕士	2020级	周东明	脱细胞基质来源的海藻酸人工骨膜的制备	自然科学	临床医学（专业学位）
61	内蒙古科技大学包头医学院	S20210166Z	郎玉	硕士	2020级	苏燕	D-核糖通过加速血红蛋白糖基化促进红细胞衰亡的机制研究	自然科学	生物学（学术学位）

62	内蒙古科技大学包头医学院	S20210167Z	孙悦	硕士	2020级	戈娜	基于FXR信号通路探讨熊果酸对酒精性损伤大鼠的保护作用	自然科学	公共卫生与预防医学（学术学位）
63	内蒙古科技大学包头医学院	S20210168Z	刘倩	硕士	2020级	李旻辉	干旱胁迫下黄花软紫草幼苗的代谢组学和转录组学联合分析	自然科学	药学（专业硕士）
64	内蒙古科技大学包头医学院	S20210169Z	米淑宏	硕士	2020级	石继海	探讨高频超声对酒渣鼻诊断作用的研究	自然科学	临床医学（专业学位）
65	内蒙古科技大学包头医学院	S20210170Z	韩书娟	硕士	2020级	张永红	miR-21和miR-100在双酚A诱导分化型甲状腺癌进展中的表达	自然科学	临床医学（学术学位）
66	内蒙古科技大学包头医学院	S20210171Z	袁文欢	硕士	2020级	罗琳	基于结构和功能磁共振对脑震荡的研究	自然科学	临床医学（学术学业）
67	内蒙古科技大学包头医学院	S20210172Z	张秋怡	硕士	2020级	马宝慧	依赖Caspase-1经典细胞焦亡途径在氟中毒致大鼠脑损伤中的作用研究	自然科学	生物学（学术学位）
68	内蒙古科技大学包头师范学院	S20210176Z	宋雨彤	硕士	2020级	刘春玲	明代洪武到弘治年间黄河北道入海口地区的生态治理研究	人文社科	中国史
69	内蒙古科技大学包头师范学院	S20210177Z	张心阳	硕士	2020级	姜明	珍惜“模范自治区”荣誉称号推进新时代内蒙古民族工作高质量发展	人文社科	马克思主义理论
70	内蒙古科技大学包头师范学院	S20210178Z	董向阳	硕士	2020级	刘利	包头南海子湿地自然保护区鸟类群落结构及多样性研究	自然科学	生物学
71	内蒙古科技大学包头师范学院	S20210179Z	郝志军	硕士	2020级	赵建军	Pr0.87-xGdxCa0.13MnO3的电磁特性研究	自然科学	物理学
72	内蒙古工业大学	S20210180Z	崔昊	硕士	2020级	崔晓明	微量元素Cr对ZL101铝合金的微观组织及力学性能和耐腐蚀性能的影响研究	自然科学	材料与化工（材料工程）
73	内蒙古工业大学	S20210181Z	陈嘉炜	硕士	2020级	高晓平	复合装甲用碳/芳混杂纤维增强复合材料性能研究	自然科学	材料与化工（纺织工程）
74	内蒙古工业大学	S20210182Z	王云辉	硕士	2020级	张建伟	基于矩阵变换器的飞轮储能模型预测控制	自然科学	电力电子与电力传动
75	内蒙古工业大学	S20210183Z	郄佳婷	硕士	2020级	张健欣	微通道内非接触式粒子测温结构的设计实现	自然学科	控制科学与工程
76	内蒙古工业大学	S20210184Z	张甜甜	硕士	2020级	刘培玲	极端高磁场对不同含水量淀粉结构性质及功能的影响研究	自然科学	化学工程与技术
77	内蒙古工业大学	S20210185Z	冯会聪	硕士	2020级	王红	抗烧结高效CO_2甲烷化催化剂的制备及催化机理研究	自然科学	化学工程与技术
